# Research and Application of Safety Hazard Perception and Responsibility Traceability System in University Laboratories

**DOI:** 10.3390/s26030953

**Published:** 2026-02-02

**Authors:** Rundong Liu, Yuxuan Ding, Xiujin Zhu, Xin Xia

**Affiliations:** School of Environmental Science and Engineering, Suzhou University of Science and Technology, Suzhou 215009, China; 13914770348@163.com (Y.D.); 22200130219@post.usts.edu.cn (X.Z.); 15251942091@163.com (X.X.)

**Keywords:** university laboratories, perception of potential safety hazards, retrospective liability, machine learning, target tracking

## Abstract

In order to solve the challenges of laboratory safety management in universities, such as insufficient supervision of high-frequency risk behaviors in responsibility traceability, a laboratory safety hazard perception and responsibility traceability system based on deep learning is proposed. Based on the YOLOv5s object detection model, the channel attention mechanism SE and NWD loss functions are introduced, with DeepSORT tracking to realize multi-target tracking and hidden danger perception in laboratory scenarios. Then, the responsibility matching algorithm and visual traceability mechanism are proposed to build a full-chain management system of “risk perception, analysis and tracking, and responsibility traceability”. Experiments show that the mean average precision (mAP) of YOLO-lab in the laboratory scene is 87.8%. Taking the experimenter not wearing a lab coat as an example, through the test of the laboratory scene, the multi-target tracking effect is excellent and the responsibility traceability report is generated, which solves the problem of “visible and uncontrollable behavior, traceable and unproven” in traditional supervision, and provides an intelligent technical path for laboratory safety governance.

## 1. Introduction

As the core field of talent training and scientific research innovation [1,2,3,4], the intelligent upgrading of the safety management system of university laboratories has become a key proposition to ensure the orderly development of scientific research activities [5,6,7,8]. In recent years, the development of Internet of Things technology has provided a foundation for active prevention and control of laboratory safety [9,10]. Especially in the field of intelligent management of university laboratory safety, multi-level and multi-technology integration solutions have been formed. Based on multi-sensor data fusion, wireless communication, and cloud platform technology, Wu Leyuan et al. built a three-layer architecture including perception layer, transmission layer, and application layer, realizing real-time collection, multi-level early warning, and automatic intervention of laboratory environmental parameters [11]. Yang Yan et al. took STC89C52 microcontroller as the core, integrated fingerprint recognition, temperature, humidity, and smoke sensors, and built a localized security management system with authority authentication, environmental monitoring, and abnormal alarm functions [12]. From the perspective of computer risk identification, Deng Tian analyzed the potential safety hazards of the laboratory in terms of equipment, network, and human operation and put forward systematic safety management countermeasures [13]. Shu Qiang et al. combined the Internet of Things and situation-aware computing to construct an emergency decision-making mechanism based on the fusion of meta-rules and knowledge, which significantly improved the response speed and disposal accuracy of laboratory safety accidents [14].

However, at the level of normalized supervision involving personnel code of conduct, the prior art still lacks in the perception and management of high-frequency and risky behaviors of experimental operators, especially in the subsequent determination of responsibility. High-frequency risk behaviors (such as not wearing experimental protection for experimental operations, illegal carrying of unstandardized labeled bottles, etc.) have strong randomness and low-risk perception characteristics, and their regulatory difficulties lie in the superposition effect of personnel behavior heterogeneity and environmental complexity in laboratory scenarios. This leads to the fact that the daily safety management of the laboratory relies on manual inspection and post-event accountability, which is often inefficient, which is a key issue that must be considered in the future construction of high-quality smart laboratories.

With the breakthrough of computer vision technology, object detection algorithms based on deep learning provide a new path for building an active security protection system [15]. With its end-to-end detection architecture and real-time reasoning capabilities, YOLO series algorithms have shown significant advantages in the field of industrial safety monitoring [16,17,18,19], such as the optimization case of recognition and helmet wearing detection [20,21,22,23], which confirms the adaptability of this technology to complex dynamic scenarios.

At the same time, as a visual object tracking algorithm based on deep learning, DeepSORT combines deep learning and traditional object tracking algorithm SORT, which has high accuracy, strong robustness, and good adaptability to target occlusion, deformation, and other situations [24]. It has been widely used in the fields of tracking pedestrians, vehicles, and other targets and intelligent video surveillance; usually, DeepSORT detects the target in each frame of the image based on object detectors, such as YOLO, Faster R-CNN, etc., and there are many cases of YOLO + DeepSORT tracking people in different scenarios, such as subway corridors [25]. Its purpose is to count and monitor unsafe behaviors of personnel in real time, but in the university laboratory scenario, there are few cases of using YOLO + DeepSORT for tracking unsafe behaviors of personnel, so this paper uses YOLO-lab + DeepSORT to achieve efficient personnel tracking in university laboratory scenarios.

In this study, the object detection and multi-object tracking technology are combined as the technical base of responsibility matching algorithm and process traceability, and an integrated system of real-time safety perception monitoring and responsibility traceability is built in the laboratory. The system design breaks through the traditional monitoring or “supervision separation” safety management model and realizes a new management model from risk perception to process tracking to responsibility traceability.

The rest of this paper is organized as follows. Section 2 will introduce the overall architecture of the safety hazard perception and responsibility traceability system, detailing its layered design and functional modules. Section 3 will elaborate on the key technologies employed in the system, including the improved YOLO-lab detection model, the DeepSORT-based personnel tracking algorithm, and the responsibility matching and process traceability mechanisms. Section 4 will present experimental results and application cases, demonstrating the system’s performance in real laboratory environments. Finally, Section 5 will summarize the research findings and propose directions for future work.

## 2. Design Architecture of Safety Hazard Perception and Responsibility Traceability System

**Figure 1:**
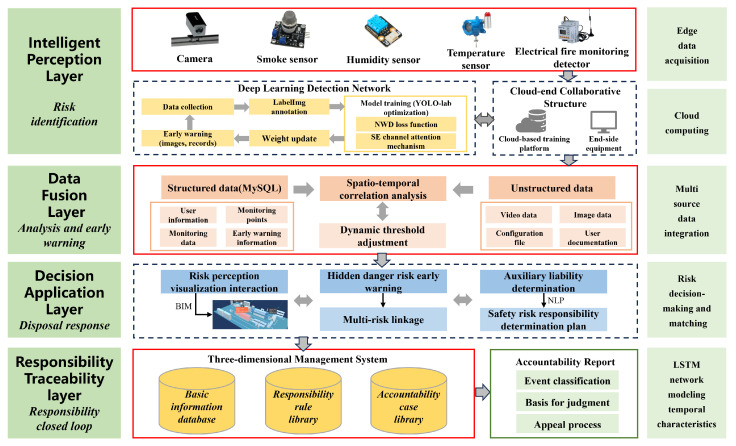
The architecture of laboratory safety hazard perception and responsibility traceability system.

According to the characteristics of high-frequency risk behavior in university laboratories, the design architecture of the risk perception and responsibility traceability system is shown in Figure 1. The system adopts a hierarchical and progressive structure, consisting of an intelligent perception layer, a data fusion layer, a decision-making application layer, and a responsibility traceability layer, forming a full-chain management system of “risk identification, analysis and early warning, disposal response, and responsibility traceability”.

The intelligent perception layer relies mainly on edge data collection and the deep learning detection network to build a laboratory security risk perception detection network, and the algorithm deployment adopts a collaborative cloud-edge-end architecture. Localized model inference is carried out at edge computing nodes, and low-latency early warning is combined with cameras to adapt to the deployment needs of complex laboratory environments to realize the identification and perception of potential safety hazards.

The data fusion layer builds a multi-source heterogeneous data integration framework, integrating video streams, sensor monitoring data, and environmental parameters through threshold analysis, spatio-temporal correlation and other technologies, uses the MySQL database to achieve structured data storage, and combines unstructured data management modules to establish a multi-source data fusion laboratory safety hazard database.

The decision-making application layer includes three modules: risk perception visual interaction, the early warning of hidden danger risk, and auxiliary responsibility determination, which uses natural language generation technology to automatically generate security risk responsibility determination plans and provides hierarchical response strategies based on matching algorithms to achieve dynamic decision-making from single risk early warning and disposal to multi-risk linkage.

The basic information database includes the information of the laboratory leader, the list of access laboratories, the information of instrument users, etc. The responsibility judgment rule database is determined according to the specific requirements of laboratory safety supervision, and the accountability case database is used to use the data of daily safety inspection and hidden danger rectification process as the historical database of safety events through the built-in responsibility matching algorithm and process traceability technology. The system automatically generates the basic information of the event. The responsibility traceability report of incident evidence cooperates with the appeal process to form a closed loop of responsibility traceability.

## 3. Safety Hazard Perception and Responsibility Traceability System Technology

The accurate perception and dynamic tracking of laboratory safety risks are the core technical foundation for building an intelligent governance system, and its performance directly determines the timeliness of early warnings for potential hazards, the credibility of responsibility traceability, and the overall robustness of the system. This system adopts the time series architecture that integrates the YOLO-lab deep detection network and DeepSORT multi-object tracking algorithm, building a new paradigm of target tracking for the detection of potential safety hazards in the laboratory by introducing the channel attention mechanism to optimize the loss function, enhance the target characteristics, and improve the detection performance of small objects [26].

### 3.1. The Model of Risk Perception and Detection for Laboratory Safety Based on YOLO-Lab

**Figure 2:**
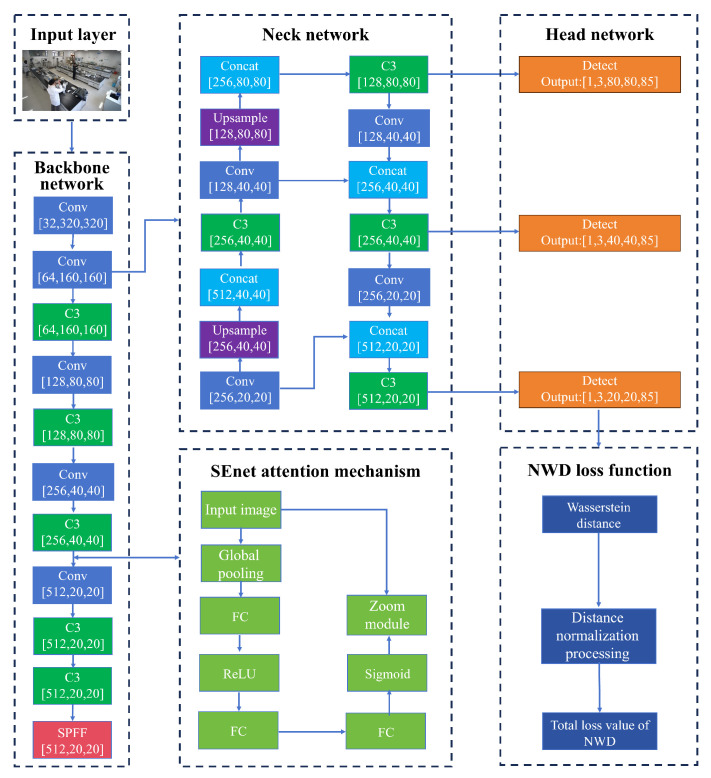
The architecture of YOLOv5 multi-scale object detection network.

According to the characteristics of dense laboratory personnel and remarkable fine-grained characteristics of protective equipment, a heterogeneous feature enhancement network based on YOLOv5s is constructed, as shown in Figure 2 [27]: Dynamic Mosaic-9 data enhancement technology is introduced at the input end to expand the sample diversity and improve the average accuracy of small target detection through the nine-square grid splicing strategy [28]. The dual improvement mechanism of backbone network fusion is as follows: (1) embed the channel attention module SE between the C3 layer and the fully connected layer to establish the characteristic channel weight distribution to strengthen the characteristic response of the people in the area wearing the experimental protective clothing; (2) using the NWD loss function and using the similarity between the distribution of Wasserstein distance measurements, the problem of unregulated label bottles being difficult to detect when far away from the camera is a small target.

#### 3.1.1. Improvements in Attention Mechanisms

**Figure 3:**
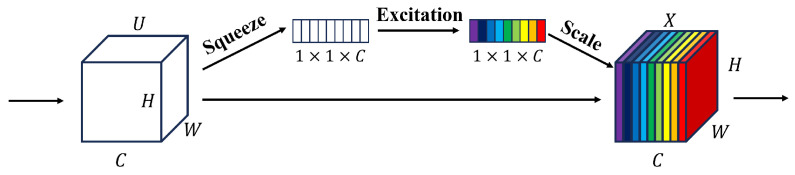
YOLO-lab algorithm to compress the activation module.

In the complex scenario of the laboratory, it is inevitable that the target personnel and other personnel will block each other, resulting in a decrease in the available detection characteristics, resulting in missed detection, false detection, and reduced detection accuracy. In this paper, the SEnet (Squeeze and Excitation Network) module based on the channel attention mechanism is inserted between the C3 layer and the fully connected layer in the backbone part of the feature extraction network. The extracted 512 channels are given different weights so as to suppress the invalid features in the channel and enhance the valid features, reducing the information redundancy and the probability of missed detection and false detection of the target. The SE module of YOLO-lab is shown in Figure 3 [29].

Among them, the compression module: through global average pooling, the two-dimensional features (H∗W) of each channel are compressed into 1 real number, and the feature map is changed from [h,w,c]==>[1,1,c]. Activate the module: generate a weight value for each feature channel and build the correlation between channels through two fully connected layers. The number of output weight values is the same as the number of channels in the input feature map. [1,1,c]==>[1,1,c]. Scaling module: Weighs the previously obtained normalized weights to the features of each channel, multiplying each channel by the weight coefficient [h,w,c]∗[1,1,c]==>[h,w,c].

#### 3.1.2. Improvements in Loss Functions

The prediction layer of YOLOv5s uses the bounding box of IoU as a loss function, which solves the problem of intersection over union (IoU), but it does not solve the problem that the performance of IoU is significantly reduced due to the slight deviation of the prediction box and the small number of pixel valuing that can be extracted from small targets [30]. In order to solve the problem that it is difficult to accurately detect small targets when the unstandardized label bottle is far away from the camera in the laboratory scenario, the NWD (Normalized Gaussian Wasserstein Distance) loss function module is introduced to solve the problem of IoU detection bias. The NWD loss function is a new method of calculating the similarity between boxes and boxes, which is based on modeling the box as a Gaussian distribution, using Wasserstein distance to measure the similarity between the two distributions, and then replacing IoU. Compared with IoU, NWD has the advantages of scale invariance, gentle differential transformation of positions, and the ability to measure the similarity of disjoint boxes.

Assuming that the bounding box R=(cx,cy,w,h), the second-order Wasserstein distance for two bounding boxes can be defined as:

(1) $$W_{\alpha }^{2}\left(N_{1},N_{2}\right)={∥\left({\left[cx_{p},cy_{p},\frac{w_{a}}{2},\frac{h_{a}}{2}\right]}^{T},{\left[cx_{q},cy_{q},\frac{w_{b}}{2},\frac{h_{b}}{2}\right]}^{T}\right)∥}_{2}^{2}$$

$W_{\alpha }^{2}\left(N_{1},N_{2}\right)$ for distance measures, which cannot be directly used for similarity measures; the normalized exponent receives a new measure: Equation (2) is the normalized Wasserstein distance [31]:

(2) $$NWD\left(N_{1},N_{2}\right)=\exp\left(-\frac{\sqrt{W_{\alpha }^{2}\left(N_{1},N_{2}\right)}}{C}\right)$$

where *C* is the constant related to the dataset, and then the loss function based on NWD in Formula (3) is obtained

(3) $$L_{NWD}=1-NWD\left(N_{1},N_{2}\right)$$

Compared with the IoU loss function in the YOLOv5s architecture, NWD can obtain similarity measurements in the face of overlapping targets, and the recognition of small targets such as unstandardized label bottles in laboratory scenarios is more efficient, improving the detection accuracy of small targets with a certain convergence speed.

### 3.2. Personnel Tracking Algorithm Based on DeepSORT

**Figure 4:**
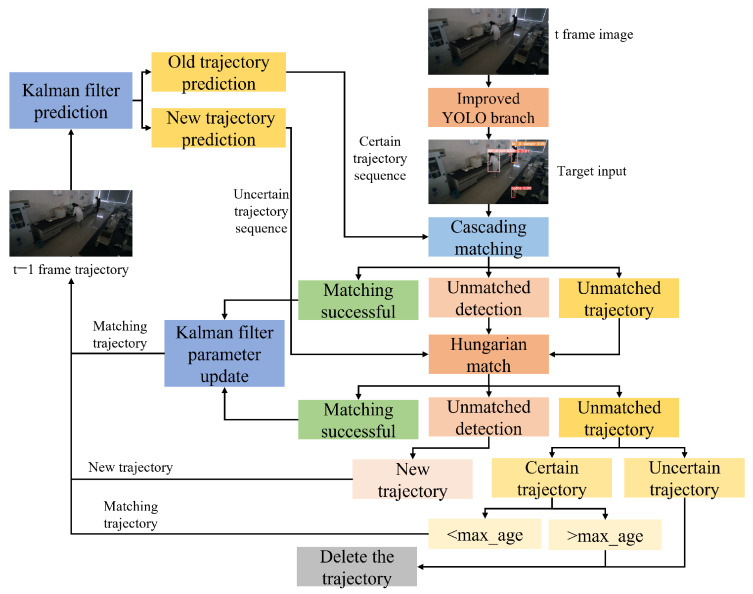
The flowchart of the DeepSort algorithm.

In order to obtain real-time information on the activities of personnel in the laboratory, this paper uses the DeepSORT algorithm proposed by Wojkerz [32] to track and locate the personnel in the laboratory. The process of the DeepSORT algorithm is shown in Figure 4, which introduces cascade matching based on the SORT (Simple Online and Realtime Tracking) algorithm [33]. The correlation process between the prediction and detection frames of the matching target is used by the Kalman filter and the Hungarian algorithm [34], and the Intersection over Union (IoU) between the tracking result and the detection result is used as the evaluation criterion for the similarity between the prediction bounding box and the detection bounding frame [35], thus reducing the occlusion problem [36].

In the process of tracking laboratory personnel activities, DeepSORT uses the 8-dimensional variable *t* to express the appearance and movement information of the relevant experimenters, represented by Equation (4).

(4) $$t=(x,y,z,u,\dot{x},\dot{y},\dot{z},\dot{u})$$

where (x,y) is the central goal of the experimenter; *z* is the width ratio of the personnel detection box; *u* is the height of the personnel detection box, $\left(\dot{x},\dot{y},\dot{z},\dot{u}\right)$ is the speed information corresponding to (x,y,z,u).

DeepSORT combines the information of the relevant personnel in the laboratory to correlate the data, link the target information, and use the predicted Kalman filter. The Mars distance between the measured value is used to deduce the test results, as shown in Equation (5).

(5) $$d^{\left(1\right)}\left(i,j\right)={\left(d_{j}-y_{i}\right)}^{T}S_{i}^{-1}\left(d_{j}-y_{i}\right)$$

where $d_{j}$ is the position of the *j*th detection frame, $y_{i}$ is the predicted position of the *i*th relative target, and $S_{i}$ indicates the covariance matrix between the detection position and the average tracking position. To ensure the stability of the prediction results, DeepSORT applies a threshold for the Mars distance based on a 95 % confidence interval, as shown in Equation (6).

(6) $$c^{\left(1\right)}\left(i,j\right)=1\left[d^{\left(1\right)}\left(i,j\right)\leq t^{\left(1\right)}\right]$$

where $t^{\left(1\right)}\;$ is the Mars threshold, and for the four-dimensional measurement space, the corresponding Mars threshold is $t^{\left(1\right)}\;=9.4877$.

DeepSORT introduces a second-order association metric in the node, where the neural network extracts a cosmetic feature vector for each tracked target and determines the correlation by calculating the minimum cosine distance between the detection of the current frame and the target feature vector tracked historically, as shown in Equation (7).

(7) $$d^{\left(2\right)}\left(i,j\right)=\min\left\{1-r_{j}^{T}r_{k}^{\left(i\right)}|r_{k}^{\left(i\right)}\in R_{e}\right\}$$

where $r_{j}$ is the eigenvector corresponding to the detection box; $r_{k}$ is the eigenvector that has been successfully associated.

The movement and appearance of laboratory personnel are obtained by linear weighting to form Equation (8) as the final index, and then the final cost matrix related to the Hungarian algorithm is calculated, so as to achieve accurate and real-time multi-object tracking in the laboratory scenario.

(8) $$f_{i,j}=\lambda d^{\left(1\right)}\left(i,j\right)+\left(1-\lambda \right)d^{\left(2\right)}\left(i,j\right)$$

where λ is the weight coefficient; if $f_{i,j}$ falls within the specified threshold range, the correct association is achieved.

The $max_{age}$ parameter in Figure 4 is defined as the maximum time of existence of a target trajectory, which increases when a target trajectory does not match the detection result for multiple consecutive frames. If the existence time exceeds the $max_{age}$ setpoint, the track is flagged and subsequently deleted.

### 3.3. Implementation of Responsibility Matching and Process Traceability System

The spatio-temporal data flow generated by risk perception and dynamic tracking technology provides a technical basis for multi-dimensional correlation analysis for the responsibility matching and process traceability system [37,38]. The traditional responsibility traceability mechanism is limited by discrete evidence collection capabilities and static causal reasoning modes, which can easily lead to ambiguous attribution and scattered accountability basis for laboratory safety accidents. Therefore, the system is based on spatio-temporal trajectory modeling, multi-modal feature extraction, and dynamic rule engine to accurately track the main responsibility of personnel in laboratory safety incidents [39]. The system architecture includes two core modules, the responsibility matching algorithm engine and the process traceability workflow, forming a technical closed loop from micro process traceback to macro responsibility determination [40].

#### 3.3.1. Responsibility Matching Algorithm

**Figure 5:**
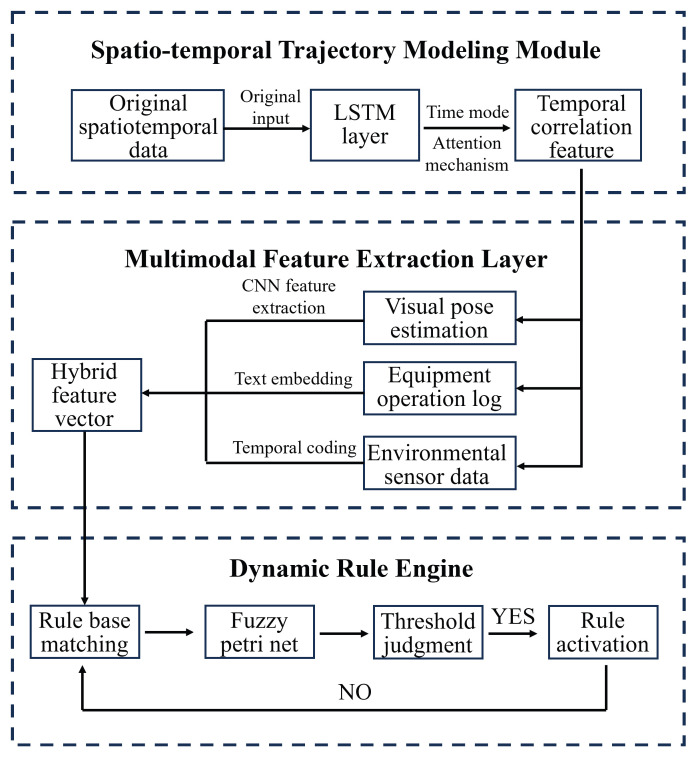
The flowchart of the responsibility matching algorithm.

The core of the responsibility matching algorithm adopts the three-level linkage reasoning architecture of “spatio-temporal trajectory-behavioral characteristics-rule base”, as shown in Figure 5. The spatio-temporal trajectory modeling module is based on the long short-term memory network LSTM, which extracts the temporal correlation characteristics of personnel’s operational behavior through the time-mode attention mechanism. The multimodal feature extraction layer integrates visual pose estimation, equipment operation logs, and environmental sensor data to construct a hybrid feature vector containing four-dimensional spatiotemporal coordinates and three types of behavior labels. The dynamic rule engine integrates the laboratory safety protocol knowledge base, adopting fuzzy Petri network modeling technology, and realizes the dynamic matching of rule reasoning and feature vectors through the transition trigger function.

#### 3.3.2. Traceability Process

**Figure 6:**
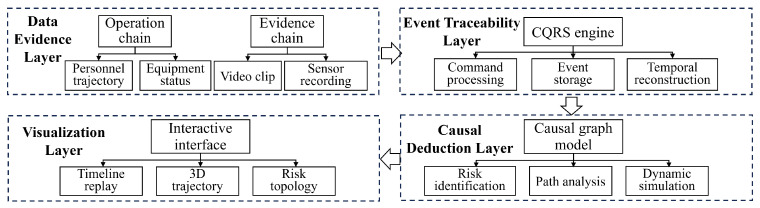
The flowchart of the traceability process.

The traceability process workflow adopts the chain architecture of “data storage, event traceability, causal deduction, and visualization”, as shown in Figure 6. The data storage module is designed with a dual-chain storage structure: the operation chain solidifies process data such as personnel spatio-temporal trajectories and equipment state changes, and the evidence chain stores key evidence such as video clips and sensor alarm records. The event traceability engine is based on the CQRS (Command Query Separation of Responsibilities) mode, and the operation timing is reconstructed through the event playback function, while the causal deduction combines the causal graph model to analyze the risk transmission path, and uses interactive functions such as visual interface display and multi-perspective operation playback to realize the visual verification of the responsibility traceability process.

## 4. Experiment and Application

### 4.1. YOLO-Lab Improved Algorithm Experiments

#### 4.1.1. Hardware System

The hardware system features a modular suspended track structure. Its dual-linear inspection track design fulfills the core requirements of high-precision positioning, low-interference operation, and rapid deployment. The system’s built-in vision module achieves full coverage of the laboratory through I-beam aluminum alloy tracks, establishing a comprehensive spatial perception network.

**Figure 7:**
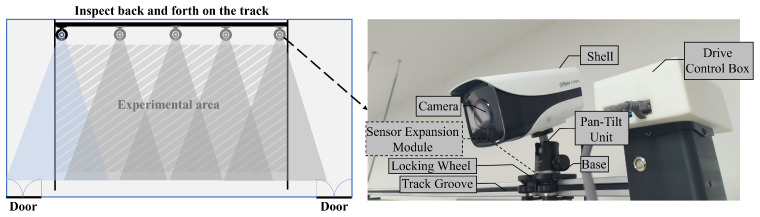
The diagram of the hardware system.

The hardware system are mainly composed of shell, camera, base, drive control box, track, gimbal, and multi-modal sensor. The environmental monitoring system integrates multiple sensors to achieve comprehensive situational awareness and safety alerts in laboratories. For smoke and hazardous gas detection, the MQ-2 gas sensor (Zhengzhou Weisheng Electronic Technology Co., Ltd., Zhengzhou, China) is employed, known for its high sensitivity and rapid response, generating output signals proportional to gas concentration levels. Temperature is monitored using the compact and noise-immune DS18B20 digital temperature sensor (Analog Devices, Inc., Shanghai, China). Humidity sensing is handled by the DHT11 sensor (Guangzhou Aosong Electronic Co., Ltd., Guangzhou, China), which utilizes a capacitive humidity sensing element to provide real-time digital output with low power consumption and cost-effectiveness. Also, the Dahua DH-IPC-HFW1235M-A-I1 wide-angle camera (horizontal resolution 1920 × 1080, frame rate 25 fps, Zhejiang Dahua Technology Co., Ltd., Hangzhou, China) is deployed to ensure that the viewing angle can fully cover the entire experimental area during the movement of the track, as shown in Figure 7.

#### 4.1.2. Experimental Environment and Dataset

The experimental environment is built based on the real scene of the university laboratory, and the edge computing unit uses NVIDIA Jetson Xavier NX (32 TOPS AI computing power, NVIDIA, Santa Clara, CA, USA) equipped with PyTorch 1.10 and TensorRT 8.2 acceleration framework. The deep learning framework is PyTorch, the experimental platform is a mobile computer, the system is Windows 64-bit, the CPU is Corei5-12400, the GPU is NVIDIA GeForce RTX 3060 (NVIDIA, Santa Clara, CA, USA), the language is Python 3.8.0, the neural network framework Pytorch, CUDA 11.8, and the epochs are set to 50, and in order to ensure the reliability of the experimental results, the model training hyperparameters are consistent throughout the experimental process.

The construction of experimental datasets follows the principle of “common benchmark + feature enhancement”, integrating public datasets such as Indoor Scene Recognition (MIT) and self-built LabDetection special datasets, and selects typical plots of experimental datasets.

**Figure 8:**
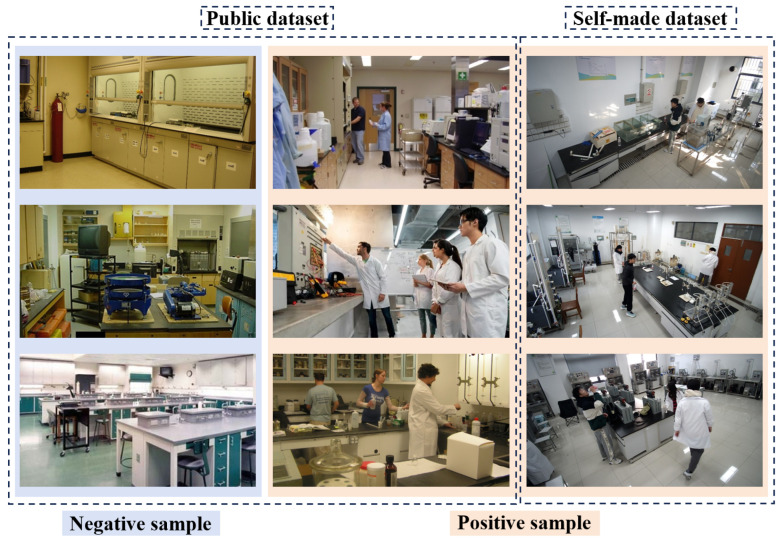
Typical diagram of an experimental dataset.

Positive samples are clearly marked as target categories (such as objects or people detected by objects in laboratory scenes), and negative samples represent background or non-target objects (such as some static scenes), and the model can accurately distinguish between targets and interference items by optimizing the feature differences between the two. Taking the monitoring of violations without wearing lab coats as an example, both “not wearing lab coats” and “wearing lab coats” are used as detection targets, and the model needs to establish a benchmark feature representation through clear positive samples, so as to enhance the discrimination ability of negative samples. Specifically, the single feature of the lab coat provides a highly discriminative feature anchor for the model, avoids the confusion of features caused by different clothing textures and colors, and enhances the generalization ability of the model to abnormal situations. Its core logic stems from the necessity of deep learning models to learn negative and positive sample features, as shown in Figure 8.

The public dataset is adapted to MIT’s Indoor Scene Recognition, which contains 67 Indoor categories with a total of 15,620 images. The number of images varies by category, but there are at least 100 images in each category. The dataset comprises 121 images selected from the relevant categories of the MIT dataset, among which 49 contain detectable targets, while the remaining 72 do not, and finally obtains 605 images by adding noise, adjusting brightness, cropping, rotating, mirroring, and other enhancement operations.

Characteristics of self-made dataset: In the laboratory of the School of Environmental Science and Engineering of Suzhou University of Science and Technology, a total of 424 images were annotated with LabelImg using multi-perspective scene data collection, and 2120 images were finally obtained after enhancement operations such as adding noise, adjusting brightness, cropping, rotating, and mirroring.

Among the total 2725 images, there are 933 instances of wearing lab coats, 662 instances of not wearing lab coats, 901 instances of abnormal reagent bottles (with one picture and multiple targets), and the personnel leaving targets are annotated by time series, and the dataset is divided according to 8:1:1 (training set: verification set: test set), and the number of labels is shown in Table 1.

#### 4.1.3. Evaluation Indicators

In this experiment, the evaluation indicators in deep learning, including recall (R), accuracy (P), mean Average Precision (mAP), and frames per second (FPS), are used as evaluation indicators for target detection items in public laboratories.

(9) $$R=\frac{TP}{TP+FN}\times 100\%$$

(10) $$P=\frac{TP}{TP+FP}\times 100\%$$

(11) $$mAP=\frac{1}{M}\sum_{k=1}^{M}AP\left(k\right)\times 100\%$$

where TP (True Positives) represents the number of positive samples that are actually positive samples and predicted to be positive samples; FP (False Positives) represents the number of positive samples that are actually predicted to be negative samples; FN (False Negatives) represents the number of positive samples that are actually negative samples; AP (Average Precision) is the average accuracy; M is the number of samples tested.

Recall (R) is used to measure the model’s ability to recognize positive samples, i.e., the proportion of positive samples that are actually positive and correctly predicted. Precision (P) is used to measure the accuracy of the model’s predictions, that is, the proportion of the predictions that are positive samples and the actual positive samples; mean Average Precision (mAP) is an index that measures the average accuracy of a model in different categories and is used to comprehensively evaluate the performance of a model in each category. FPS refers to the number of frames processed per second, which is a key indicator to evaluate the speed of the object detection algorithm.

#### 4.1.4. Comparative Experiment of Target Detection Algorithm

As a classic single-stage object detection network, SSD is a typical representative of the two-stage object detection model, which is widely used in engineering practice with good detection effect and speed advantages. Table 2 shows that the accuracy of the SSD and Faster R-CNN algorithm is 25.6% and 32.4% lower than that of YOLO-lab algorithm, and the recall rate is 14.4% and 16.6% lower than that of the YOLO-lab algorithm, respectively. In terms of detection speed values, the FPS values of SSD algorithm and Faster R-CNN algorithm are 7.1% and 1.0% of YOLO-lab, respectively, which is much slower than YOLO-lab’s detection speed, so the YOLO-lab algorithm ensures both detection accuracy and detection speed.

**Figure 9:**
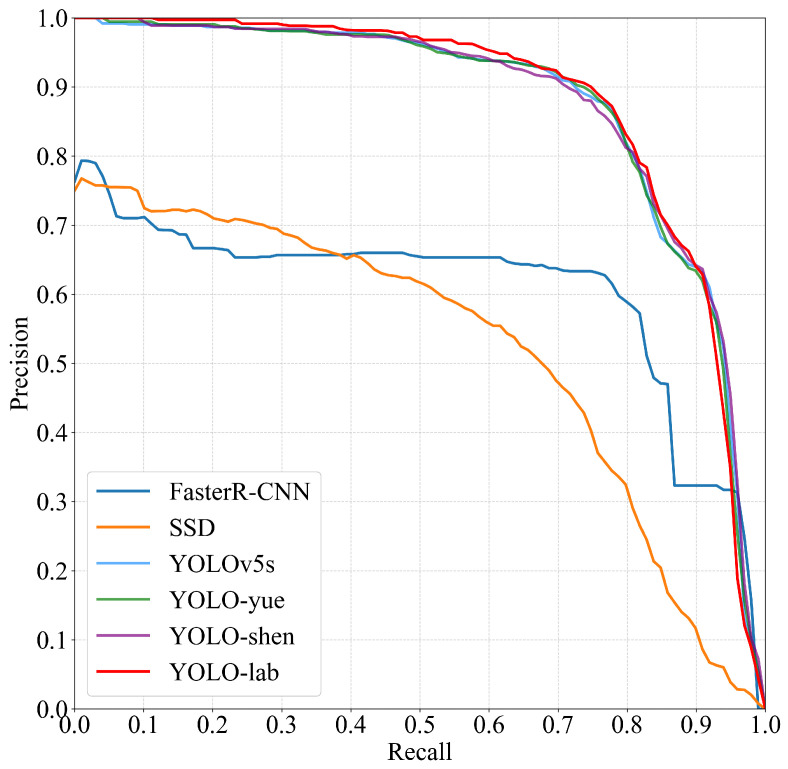
P–R curves of different models.

At the same time, in order to verify the performance of the proposed model, the improved model based on YOLOv5s by Yue Xusheng [41] and Shen Bin [42] was selected for comparison in the YOLO algorithm, and the overall algorithm accuracy was reduced by 1.8% compared with that of YOLO-lab when the detection layer was not changed. The overall accuracy of YOLO-shen is 2.4% lower than that of YOLO-lab, and the recall rate is equivalent to that of YOLO-lab, indicating that the YOLO-lab algorithm proposed in this paper takes into account the accuracy and speed of detection and shows the best performance, as shown in Figure 9.

**Figure 10:**
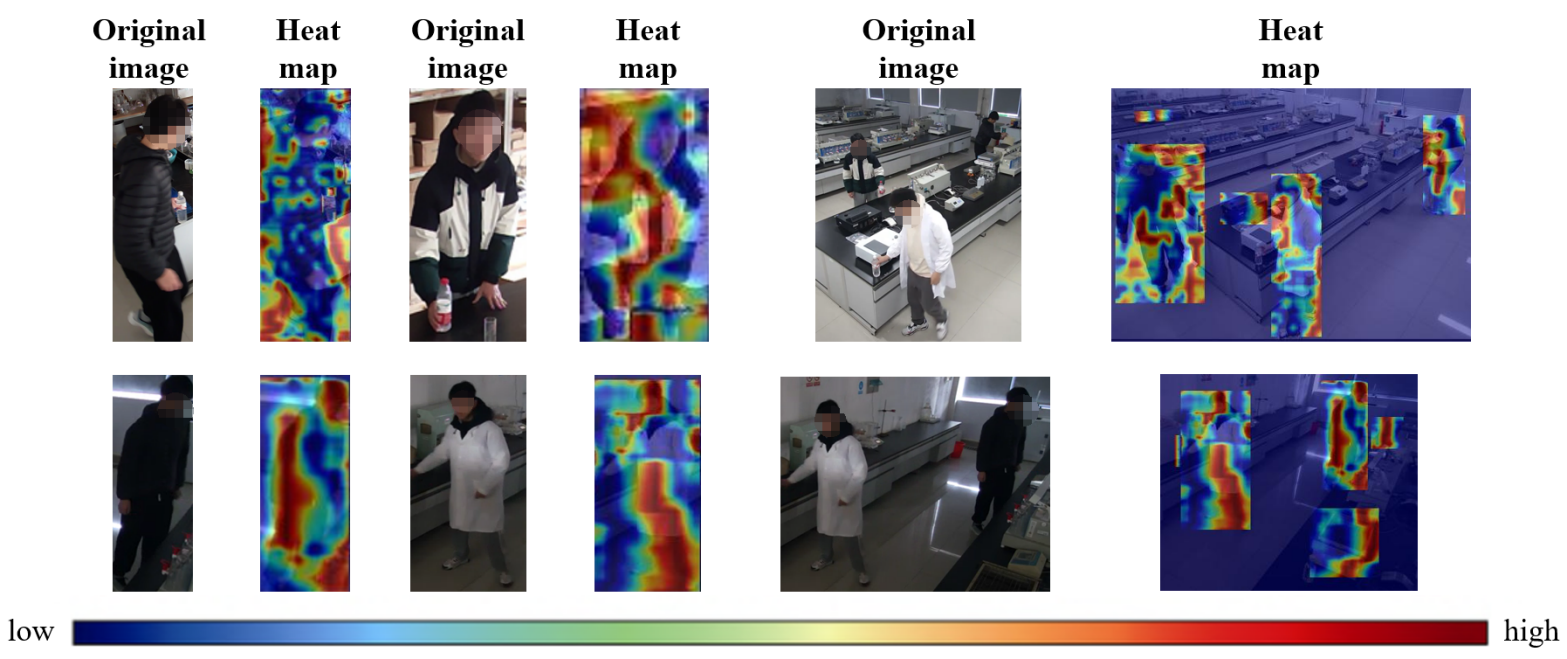
Characteristic heat map visualization.

The original version of YOLOv5s already has good detection accuracy and recall, as shown in Table 3, after embedding the SE attention module alone, the average accuracy decreases by 0.4%, but the recall rate increases by 1.2%, and the accuracy increases by 0.1% after improving the loss function NWD alone. The average accuracy of YOLO-lab proposed in this paper increased by 0.4%, the average accuracy increased by 0.5%, and the FPS decreased by 2%. As shown in Figure 10, the degree of attention the model pays to different areas is shown by color coding (blue to red). The red area is concentrated on the monitoring target, indicating that the model can effectively capture key information. The blue area corresponds to a low-concern area (such as a background or extraneous object), verifying the model’s ability to detect small targets.

**Figure 11:**
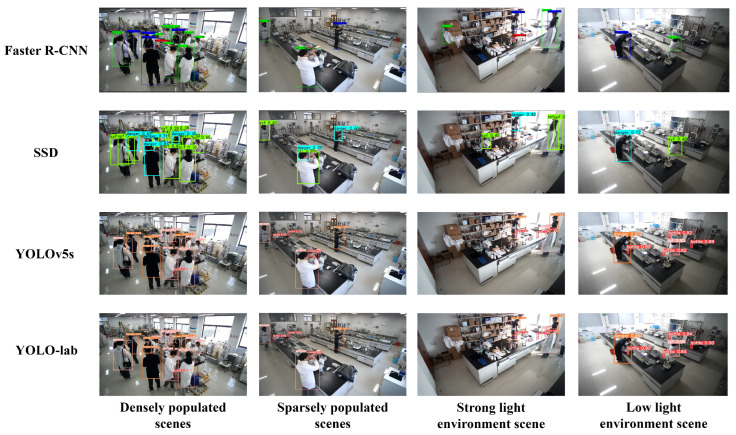
The effect of different models’ detection.

The results are shown in Figure 11. The average confidence of R-CNN is 0.775, while the average confidence of SSD is 0.6, and there are many missed detections, while YOLOv5s has excellent detection performance in four complex scenarios, and the average confidence is lower than that of the YOLO-lab algorithm proposed in this paper on individual small targets and when the light environment is weakened, and there is a possibility of missing targets. The YOLO-lab model effectively solves the problem of complex background interference, indicating that it has better detection performance in complex environments.

### 4.2. DeepSORT Personnel Track Experiments and Analysis

**Figure 12:**
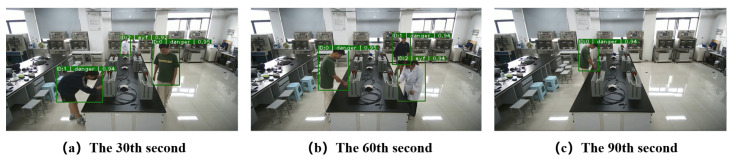
Multi-target tracking effect.

As shown in Figure 12, in the 30th second, the algorithm has tracked three targets in the scene and displayed their ID numbers, and in the 60th second, the position of the three targets in the physical space changes, and the algorithm still maintains the tracking of the three targets and the locking of the ID number, and in the 90th second, the number of targets is reduced from three to one, and the algorithm still maintains the tracking of the target and the locking of the ID number. The experiment shows that the DeepSORT personnel tracking algorithm can perform stable detection and tracking and lock numbers, which shows that it has good robustness in complex environments.

### 4.3. Application of Laboratory Hazard Perception and Traceability System

**Figure 13:**
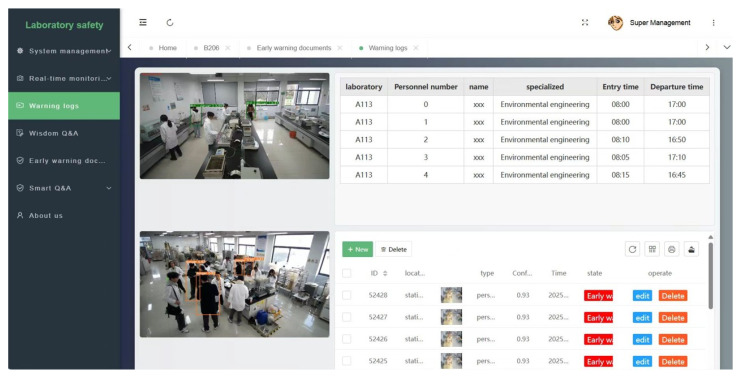
System platform diagram.

**Figure 14:**
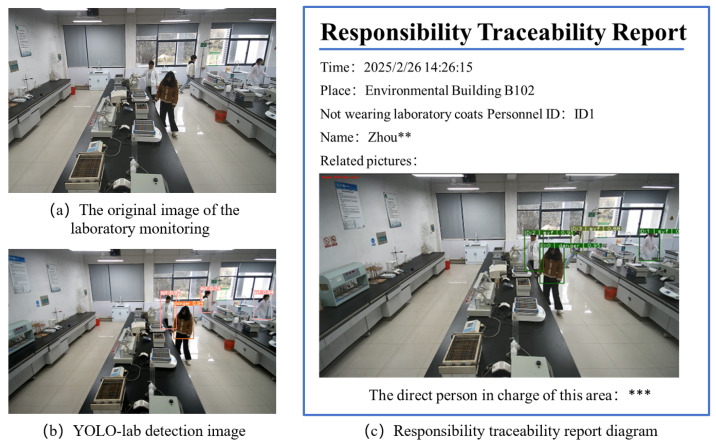
Schematic diagram of system application.

The laboratory hazard perception and traceability system collects video stream data in real time through the wide-angle camera deployed in the laboratory, combined with the machine learning object detection algorithm based on the improved YOLO-lab to identify high-frequency risk behaviors such as not wearing lab clothes and carrying unstandardized label bottles in violation of regulations, and uses the DeepSORT personnel tracking algorithm to realize the identity coding and time series tracking of laboratory personnel. In the system platform interface, as shown in Figure 13, the database module updates laboratory information in real time, including laboratory number, basic information of relevant personnel, and entry and exit time, etc., to structure data storage when the platform detects high-frequency risk behaviors, as shown in Figure 14a. In the original image of laboratory monitoring, the system background is equipped with the YOLO-lab identification module proposed in Section 3.1 for risk perception, as shown in Figure 14b. Based on the above-mentioned responsibility matching algorithm and process traceability process, the system generates a responsibility traceability report, as shown in Figure 14c. In order to consider privacy issues, we will mosaic the names of those who violate the laboratory’s regulations and the direct person in charge of the area in the report. The laboratory administrator can carry out the next step of accountability treatment according to the evidence provided in the report, according to the method of responsibility attribution, and the application presents a closed loop of safety management from “risk identification, analysis and early warning, disposal response, and responsibility traceability”.

## 5. Conclusions

This paper proposes a solution based on deep learning and Internet of Things technology to solve the problem of risk perception and responsibility traceability in university laboratory safety management. The system realizes the security perception of high-frequency risk behavior in university laboratories by improving the YOLO detection algorithm and multimodal data fusion and completes the dynamic tracking of personnel behavior based on the DeepSORT target tracking algorithm. Experiments show that the detection accuracy mAP value of the improved YOLO-lab model in complex scenarios reaches 87.8%, and the combination of responsibility matching algorithm and visual traceability mechanism realizes the security closed-loop management of university laboratories. The practical application of this system explores the transformation of laboratory safety management from passive response to active prevention and control, improves the reliability and interpretability of supervision efficiency and accident traceability, and provides the possibility of refined management for the construction of smart laboratories.

## Figures and Tables

**Table 1:** The number of labels in the training set, validation set, and test set.

Training Sets	Validation Sets	Test Sets	Total Number
3715	455	495	4655
2610	320	380	3310
3565	500	440	4505

**Table 2:** Comparison results of different models.

Models	Precision	Recall	mAP	FPS
SSD	63.2	68.8	58.3	7
Faster R-CNN	56.4	66.6	66.4	1
YOLOv5s	88.4	84.0	87.3	**101**
YOLO-yue	87.0	84.3	87.4	99
YOLO-shen	86.4	**84.9**	87.7	100
YOLO-lab	**88.8**	83.2	**87.8**	99

**Table 3:** Ablation experiment.

Models	Precision	Recall	mAP	FPS
YOLOv5s	88.4	84.0	87.3	101
YOLOv5s + SE	88.5	**85.2**	87.7	**102**
YOLOv5s + NWD	88.0	83.2	87.0	97
YOLOv5s + NWD + SE(YOLO-lab)	**88.8**	83.2	**87.8**	99

## Data Availability

Data is contained within the article.
